# Synthesis and Cytotoxic Activity of New Thiazolopyrimidine Sugar Hydrazones and Their Derived Acyclic Nucleoside Analogues

**DOI:** 10.3390/molecules25020399

**Published:** 2020-01-18

**Authors:** Ebtesam A. Basiony, Allam A. Hassan, Zahra M. Al-Amshany, Ahmed A. Abd-Rabou, Adel A.-H. Abdel-Rahman, Nasser A. Hassan, Wael A. El-Sayed

**Affiliations:** 1 Faculty of Science, Chemistry Department, Menoufia University, Shibin EL-Kom 32511, Egypt; ebtesambasiony@gmail.com (E.A.B.); adelnassar63@yahoo.com (A.A.-H.A.-R.); 2 Faculty of Science, Chemistry Department, Suez University, Suez 43511, Egypt; ahassan@su.edu.sa; 3 Applied Medical Science, Medical Laboratories Department, Shaqra University, Shaqra 11961, Saudi Arabia; 4 Department of Chemistry, Faculty of Science, King Abdulaziz University, Jeddah 21551, Saudi Arabia; zalamshany@hotmail.com; 5 Hormones Department, Medical Research Division, National Research Centre, Giza 12511, Egypt; ahmedchemia87@yahoo.com; 6 Stem Cells Lab, Center of Excellence for Advanced Sciences, National Research Centre, Giza 12511, Egypt; 7 Pharmaceutical Science Department, College of Pharmacy, Shaqra University, Shaqra 11961, Saudi Arabia; 8 Photochemistry Department, National Research Centre, Giza 12511, Egypt; 9 Department of Chemistry, College of Science, Qassim University, Buraidah 51452, Saudi Arabia

**Keywords:** thiazolopyrimidine, 1,3,4-oxadiazole, sugar hydrazones, anticancer, acyclic nucleosides

## Abstract

New thienyl- or chlorophenyl-substituted thiazolopyrimidine derivatives and their derived sugar hydrazones incorporating acyclic d-galactosyl or d-xylosyl sugar moieties in addition to their per-O-acetylated derivatives were synthesized. Heterocyclization of the formed sugar hydrazones afforded the derived acyclic nucleoside analogues possessing the 1,3,4-oxadiazoline as modified nucleobase via acetylation followed by the cyclization process. The cytotoxic activity of the synthesized compounds was studied against human breast cancer MCF7 and MDA-MB-231 cell lines as well as human colorectal cancer HCT 116 and Caco-2 cell lines. High activities were revealed by compounds **1**, **8**, **10**, **11**, and **13** against Caco-2 and MCF7 cells in addition to moderate activities exhibited by other compounds against HCT116 or MDA-MB-231 cells.

## 1. Introduction

The increased risk of cancer leading to a high mortality rate is one of the important factors that stimulates scientific research in the field of medicinal chemistry for achieving distinct results able to face such threat. Chemotherapy represents an important strategy [1] that is frequently applied for treatment of cancer. The main objective associated with numerous approved chemotherapeutic agents [2] is the apoptosis induction of cancer cells. The research for developing novel anticancer candidates, with no or minimal side effects, is of considerable interest due to the observed toxicity of current drugs towards normal cells, the suppressed drug activity and the induced drug resistance which usually lead to insufficiency in the treatment process.

Thiazolopyrimidine is one of the most interesting heterocyclic scaffolds possessing structural similarity to 5-fluorouracil (5-FU)—the well-known cancer metabolite. In addition, they have been reported to possess various important potent activities such as antimicrobial, antipsychotic, anti-inflammatory, anti-Parkinson’s, analgesic, antidepressant, anti-HIV, and anticancer activities [3,4,5,6,7,8,9,10,11]. In addition, they have been revealed with their bioactivities as transient receptor potential vanilloid–receptor 1 (TRPV1) modulators [12,13], antioxidants [14,15], pesticides [16], phosphate inhibitors [17,18], acetylcholinesterase inhibitors [19,20], and antimicrobial activities [21,22,23].

**Figure 1:**
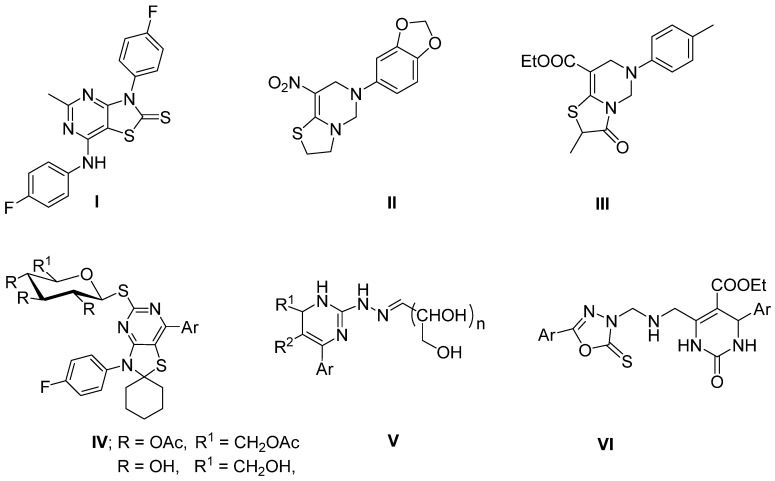
Anticancer thiazolopyrimidine, pyrinidinyl-sugar, and pyrimidinyl-oxadiazole compounds.

1,3,4-Oxadiazole is a prominent scaffold which was found to possess opulent interesting applications in drug development and designing important agrochemicals [24]. Many compounds incorporating 1,3,4-oxadiazole system showed potent bioactivities such as antiviral, anticancer, antiproliferative, antimicrobial, anti-inflammatory activities in addition to their activities as potential antifibrotic agents and monoamine oxidase B inhibitors [24,25,26,27,28,29,30,31,32]. On the other hand, acyclic and *C*-nucleoside analogs, as modified forms of natural nucleosides, have revealed important bioactivities as antibiotic, antiviral, and antitumor activities [25,26,27,33,34,35,36,37,38]. Figure 1 displays a number of thiazolopyrimidine and their incorporating sugar derivatives in addition to pyrimidine and oxadiazole hybrids possessing reported potent anticancer activities [39,40,41,42]. Recent strategies of combining various pharmacophoric scaffolds in a new hybrid structure (molecular hybridization) for constructing potent drugs have been reported to result in the formation of more potent bioactive candidates. These significances and our ongoing interest in synthesizing new active carbohydrate based heterocycles [38,43,44,45,46] prompted us to synthesize new hybrid compounds comprising thiazolopyrimidine system, aryl or thienyl moiety, and acyclic sugar or oxadiazolyl linked to sugar moiety as modified acyclic C-nucleoside analogs and studying their anticancer activity against a number of cancer cell lines.

## 2. Results and Discussion

### 2.1. Chemistry

**Scheme 1:**
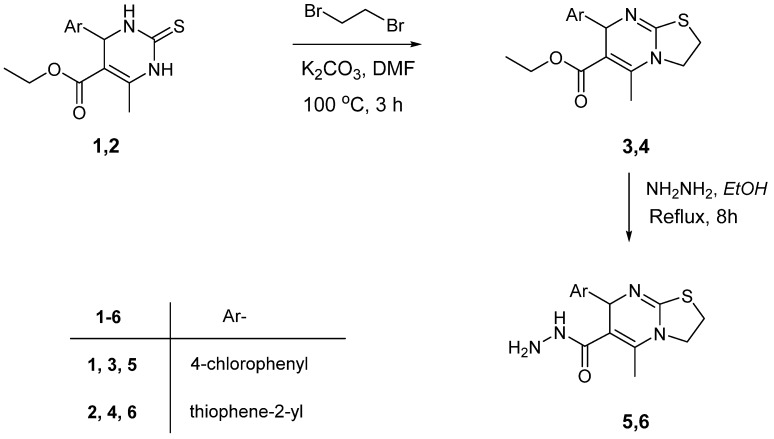
Synthesis of aryl- or heteroaryl-substituted thiazolopyrimidine derivatives.

In the present study, two types of targeted hybrid heteroaryl sugar derivatives were synthesized. The first possesses either a thienyl or chlorophenyl moiety and a thiazolopyrimidine system linked to an acyclic sugar moiety and the second incorporates an additional 1,3,4-oxadiazole system linked to acyclic sugar moiety. The substituted thiazolopyrimidine system was first prepared via a multicomponent reaction (one pot Biginelli reaction) of the aldehyde (namely; p-chlorobenzaldehyde or thiophen-2-carbaldehyde) with ethyl acetoacetate and thiourea to afford the corresponding substituted pyrimidine derivatives **1** or **2**, respectively as previously reported [47,48]. Reaction of the pyrimidine substituted ester derivatives **1** or **2** with 1,2-dibromoethane and potassium carbonate gave the corresponding thiazolopyrimidine derivatives **3** or **4**, respectively, which is similar to previously reported work [49]. Their ^1^H-NMR spectra showed the presence of the two methylene groups in addition to the characteristic triplet and quartet signals assigned for the ethyl group in addition to the aryl protons. The reaction of thiazolopyrimidine derivative **3** or **4** with hydrazine hydrate [33] gave the corresponding acyl hydrazide compound **5** or **6**, respectively (Scheme 1). Their IR spectra showed the presence of NH_2_ and NH in the region 3425–3325 cm^−1^ in addition to the characteristic carbonyl band for the amidic carbonyl group. 

The sugar hydrazone derivatives **7**–**10** were formed via the reaction of acyl hydrazides with d-galactose or d-xylose in the presence of catalytic acetic acid amount. The IR spectra of the latter sugar hydrazones revealed the bands of sugar-hydroxyl groups at 3434–3338 cm^−1^. Their ^1^H-NMR spectra revealed, in addition to the characteristic signals of the protons in the assigned structures, the H-1 methine proton at 7.20–7.45 ppm with coupling constant 8.5 Hz. The latter observed chemical shift values showed the sp^2^ hybridization of the sugar *C*-1 which indicates that the sugar moiety is present in the acyclic form.

Acetylation of the thiazolopyrimidine hydrazonyl sugar compounds **7**–**10** was achieved by means of acetic anhydride in the presence of pyridine resulting in the formation of the per-*O*-acetylated sugar hydrazones **11**–**14**, respectively. The IR spectra of the produced acetylated products revealed the existence of the C=O band of the acetyl group at 1749–1735 cm^−1^ in addition to the disappearance of OH group bands. Furthermore, their ^1^H-NMR spectra displayed the assigned signals for the protons of the five methyl groups in the CH_3_C=O group at 1.95–2.26 ppm in addition to the H-1 proton at 7.32–8.05 ppm with 7.6–7.8 Hz coupling constant, indicating the acyclic sugar form of the sugar part. 

**Scheme 2:**
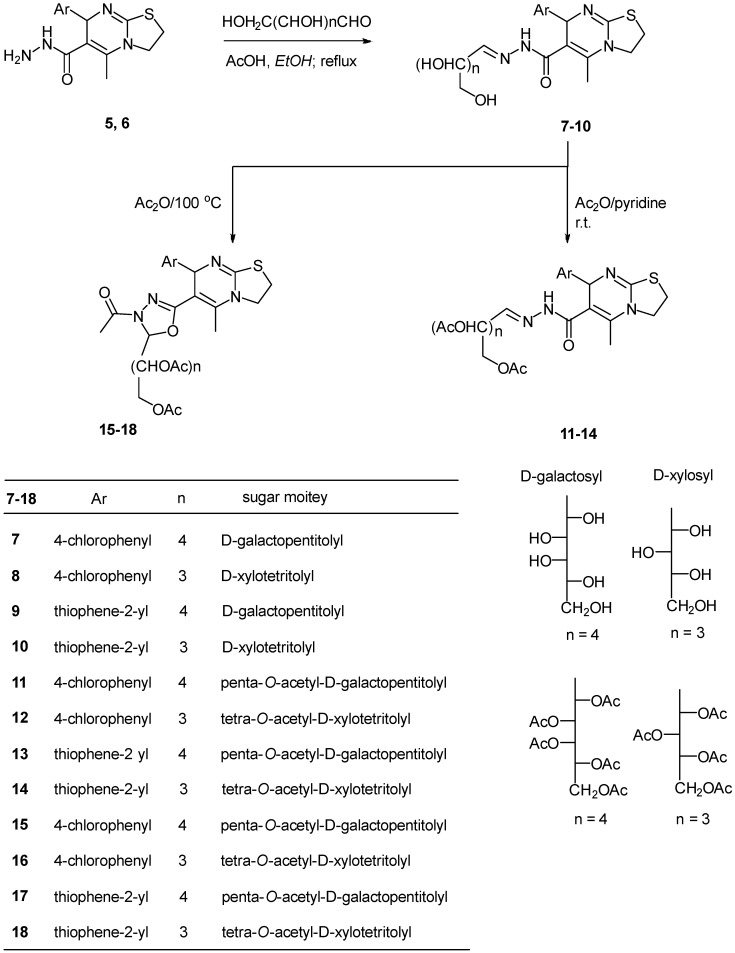
Synthesis of thiazolopyrimidine sugar hydrazones and their oxadiazolyl sugar derivatives.

On the other hand, performing the acetylation reaction of the sugar hydrazones **7**–**10** was further carried out in acetic anhydride at 100 °C and resulted in a heterocyclization process in addition to the acetylation step affording the derived 1,3,4-oxadiazoline compounds linked to acetylated acyclic sugar units **15**–**18**, respectively (Scheme 2). Formation of these oxadiazoline acyclic *C*-nucleoside analogs was consistent with the previous reported studies of the hydrazine derivatives under these conditions [24,33,38,46,50,51]. The infrared spectra of the resulting oxadiazoline sugar derivatives displayed the absorption bands attributed to the carbonyl groups of the acetyl parts at 1740–1735 and 1680–1670 cm^−1^. The signals that were afforded in their ^1^H-NMR spectra were in accordance with the assigned structures. Thus, the doublet signal at 5.70–5.72 ppm with *J* coupling 7.2–7.4 Hz corresponds to H-2 of the formed oxadiazoline ring (originally H-1 in the reacted acyclic sugar moiety) which is attached to an sp^3^ carbon atom indicating the heterocyclization process. In acyclic hydrazine forms the latter proton should be at higher chemical shift values due to the sp^2^ character of the assumed C-1 (methylenic proton). The remaining protons in the acyclic sugar skeleton were displayed at their characteristic assigned values. Furthermore, the ^13^C-NMR spectra of these products showed a signal at 81.3–82.5 ppm corresponding to the C-2 in the oxadiazoline ring (originally C-1 of the acyclic sugar part) in addition to the signals corresponding to the acetyl-carbonyl carbons and aryl carbons confirming the assigned structures. 

### 2.2. Cytotoxic Activity

In the current study, the newly synthesized compounds were examined in vitro for their cytotoxic activities against human breast cancer MCF7 and MDA-MB-231 cell lines, as well as human colorectal cancer HCT 116 and Caco-2 cell lines [52]. In addition, it will be also of interest in the present investigation to see the effect of the introduction of an acyclic sugar or oxadiazolyl linked to sugar moiety on the activity. The current results demonstrated that there was a gradual significant decrease (*p* > 0.05) of cell proliferation after treating human colorectal cancerous cell lines (HCT 116 and Caco-2) and human breast cancerous cell lines (MDA-MB-231 and MCF-7) with the synthesized compounds using different dosages started from 0 to 100 µg/mL. 

From Table 1, it has been suggested that the lower the IC_50_, the highest the cytotoxic effect against the cancer cells. Compounds which showed 100% inhibition and revealed IC_50_ values less than 100 µg/mL against at least one cancer cell line are listed in Table 1. The remaining compounds revealed undetectable IC_50_ (more than 100 ug/mL) upon all tested cancer cell lines.

**Figure 2:**
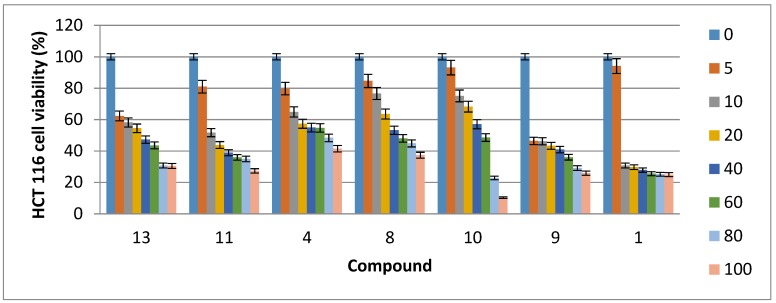
Anti-proliferative activities of compounds against human colorectal cancer HCT 116 cells. The MTT assay was performed three independent times (*n* = 3) using different concentrations of the mentioned compounds.

**Figure 3:**
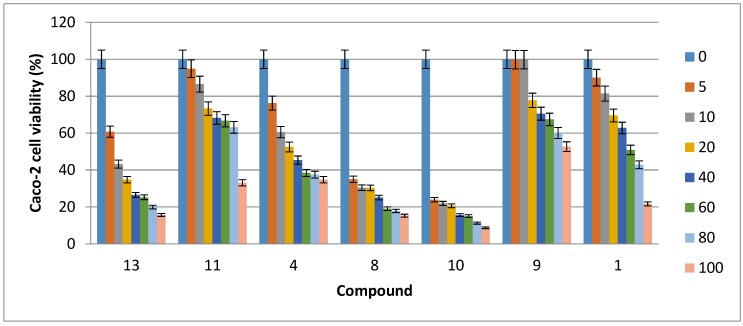
Anti-proliferative activities of compounds against human colorectal cancer Caco-2 cells. The MTT assay was performed three independent times (*n* = 3) using different concentrations of the mentioned compounds.

The observed results showed that compounds **1** and **9** exhibited the lowest IC_50_ with the highest cytotoxic effect against HCT 116 cell line with IC_50_ values 25.28 and 27.95 µg/mL, respectively. In addition, compound **11** revealed moderate cytotoxic effect against the latter cancer cell line as illustrated in (Figure 2 and Table 1). Regarding the activity against Caco-2 cell line, compounds **10**, **8**, and **13** showed the lowest IC_50_ with the highest cytotoxic effect against this cancer cell line as illustrated in Figure 3 and Table 1. 

**Figure 4:**
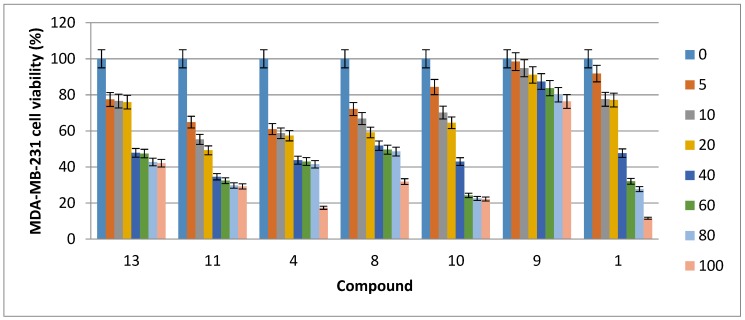
Anti-proliferative activities of compounds against human breast cancer MDA-MB-231 cells. The MTT assay was performed three independent times (*n* = 3) using different concentrations of the mentioned compounds.

**Figure 5:**
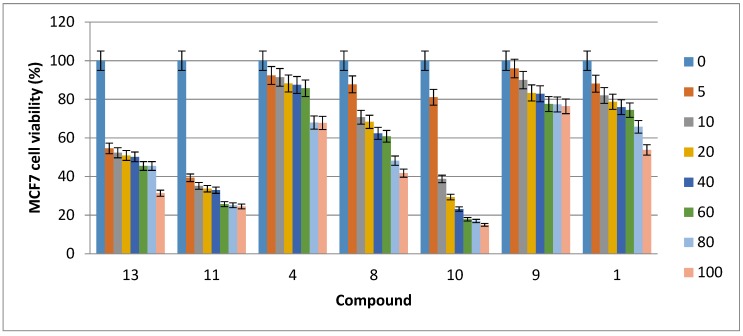
Anti-proliferative activities of compounds against human breast cancer MCF7 cells. The MTT assay was performed three independent times (*n* = 3) using different concentrations of the mentioned compounds.

On the other hand, compound **11** was shown to possess the lowest IC_50_ with the highest cytotoxic effect against MDA-MB-231 cell line as illustrated in Figure 4 and Table 1. The results also showed that compounds **10** and **4** showed moderate activities against such cancer cell line. The activity results against MCF7 cancer cell revealed that compounds **11** and **10** displayed the lowest IC_50_ with the highest cytotoxic effect as illustrated in Figure 5 and Table 1. 

By correlating of the obtained bioactivity results with the main structural features of the compounds exhibiting the highest activities, it was found that thiazolopyrimidine linked to 4-chlorophenyl or thienyl hybrid compounds incorporating acyclic sugar parts were the most active candidates. These derivatives incorporated the sugar part linked via a hydrazinyl linkage to either free hydroxyl or acetylated acyclic moiety. Thus, attachment of a hydrazinyl sugar moiety to the thiazolopyrimidine ring system (compounds **7**–**14**) resulted in higher activities compared to their starting precursors. The thiazolopyrimidine linked to acetylated galactose moiety were found higher in activities than their analogs with the five carbon xylose sugar unit. However, this was not the case for the deacetylated analogs since the free hydroxyl xylose products (**8** and **10**) were higher than those possessing galactose unit (the hydrazones **7** and **9**). The sugar hydrazones **8** and **10** with free hydroxyl xylosyl group were found higher in activities than their derived acetylated products **12** and **14**, respectively. When the sugar part was a galactosyl moiety, the acetylated derivative **11** was found higher in activity than its deacetylated analogue **7**. Furthermore, the substituted pyrimidine compound **1** was higher in its activity against HCT116 cells than the derived thiazolopyrimidine product **4** which did not incorporate sugar part. Such observations may account for the importance of the -NH linked to the thione group for the cytotoxic activity against the HCT116 cell line. However, the thiazolopyrimidine ester derivative **4** was found to be higher in the cytotoxic activity against Caco-2 cells than the substituted pyrimidine **1**. 

## 3. Experimental

### 3.1. Synthesis

#### General Procedures

Melting points were determined on a Böetius PHMK (Veb Analytik Dresden) apparatus. Thin Layer Chromatography (TLC) was performed using aluminum plates pre-coated with silica gel 60 or 60 F254 (Merck) and visualized by iodine or UV light (254 nm). The NMR spectra were recorded on a Varian Gemini 300 and Bruker DRX 400 spectrometer at 25 °C. ^1^H- and ^13^C-NMR signals were referenced to TMS and the solvent shift ((CD_3_)_2_SO δ H 2.50 and δ C 39.5). Coupling constants are given in Hz and without sign. The IR spectra (ν, cm^−1^) were recorded (KBr) on a Jasco FT/IR-410 instrument. Microanalyses were operated using Perkin Elmer 240 instrument and satisfactory results within the accepted range (±0.40) of the calculated values were obtained. All reagents and solvents were of commercial grade. The cytotoxic activity against cancer cell lines was studied at National Research Center (NRC), Dokki, Cairo, Egypt. Compounds **1** and **2** were prepared as reported previously [47,48]. 

### 3.2. Ethyl 7-(Aryl)-5-methyl-2,3-dihydro-7H-thiazolo[3,2-a]pyrimidine-6-carboxylate (***3***, ***4***)

A mixture of the ester compounds **1** or **2** (10 mmol), 1,2-dibromoethane (10 mmol), and K_2_CO_3_ (20 mmol) in DMF (12 mL) was heated in a water bath at 90 °C for 3 h, and poured on ice and cooled with water. The afforded precipitate was filtered, dried, and recrystallized from acetone–water (1:1) to give the thiazolopyrimidine derivatives **3** or **4**, respectively. 

#### 3.2.1. Ethyl 7-(4-Chlorophenyl)-5-methyl-2,3-dihydro-7*H*-thiazolo[3,2-a]pyrimidine-6-carboxylate (**3**)

Yield: 88%; 136–137 °C. IR spectrum: 3060 (C-H aromatic), 2975 (CH-aliphatic), 1695 (C=O), 1605 (C=N). ^1^H-NMR (500 MHz, DMSO-*d*_6_) δ 1.39 (t, 3H, *J* = 5.8 Hz, CH_3_), 2.11 (s, 3H, CH_3_), 3.34 (t, 2H, *J* = 5.8 Hz, CH_2_), 3.41 (t, 2H, *J* = 5.8 Hz, CH_2_), 4.12 (q, 2H, *J* = 5.8 Hz, CH_2_), 5.69 (s, 1H, H-7), 7.52 (d, 2H, *J* = 7.8 Hz, Ar–H), 7.79 (d, 2H, *J* = 7.8 Hz, Ar-H). ^13^C-NMR (DMSO-*d*_6_) δ 14.3 (CH_3_), 29.2 (CH_3_), 53.2 (CH_2_), 54.8 (CH_2_), 58.9 (CH_2_), 60.9 (pyrimidine-C), 129.6 (pyrimidine-C), 130.5 (Ar-2C), 132.5 (Ar-2C), 133.0 (Ar-C), 134.8 (Ar-C), 164.3 (pyrimidine-C), 165.5 (pyrimidine-C), 169.1(C=O). EI-MS (*m/z*, %): 336 (M^+^, 69). Anal. calcd. for C_16_H_17_ClN_2_O_2_S (336.83): C, 57.05; H, 5.09; N; 8.32. Found: C, 56.90; H, 5.02; N, 8.47.

#### 3.2.2. Ethyl 5-Methyl-7-(thiophen-2-yl)-2,3-dihydro-7*H*-thiazolo[3,2-a]pyrimidine-6-carboxylate (**4**)

Yield: 79%; 131–132 °C. IR spectrum: 3060 (C–H aromatic), 2970 (CH-aliphatic), 1670 (C=O), 1603 (C=N). ^1^H-NMR (500 MHz, DMSO-*d*_6_) δ 1.37 (t, 3H, *J* = 5.8 Hz, CH_3_), 2.10 (s, 3H, CH_3_), 3.35 (t, 2H, *J* = 5.8 Hz, CH_2_), 3.41 (t, 2H, *J* = 5.8 Hz, CH_2_), 4.12 (q, 2H, *J* = 5.8 Hz, CH_2_), 4.60 (s, 1H, H-7), 6.95–7.15 (m, 3H, thienyl-H). ^13^C-NMR (DMSO-d_6_) δ 14.6 (CH_3_), 20.3 (CH_3_), 52.5 (CH_2_), 53.9 (CH_2_), 57.9 (CH_2_), 60.5 (pyrimidine-C), 117.0 (pyrimidine-C), 127.8 (thienyl C-5), 143.2 (thienyl C-3), 143.8 (thienyl C-4), 162.9 (thienyl C-2), 165.1 (pyrimidine-C), 166.5 (pyrimidine-C), 173.3 (C=O). EI-MS (m/z, %): 308 (M^+^, 85). Anal. calcd. for C_14_H_16_N_2_O_2_S_2_(308.41): C, 54.52; H, 5.23; N, 9.08. Found: C, 54.36; H, 5.32; N, 8.95.

### 3.3. 5-Methyl-7-(aryl)-2,3-dihydro-7H-thiazolo[3,2-a]pyrimidine-6-carbohydrazide (***5***, ***6***)

A solution of the thiazolopyrimidine ester compound **3** or **4** (10 mmol) and hydrazine hydrate (30 mmol) in ethanol (25 mL) was heated under reflux for 8 h. The solution was cooled, and the resulting precipitate was filtered and recrystallized from ethanol to give **5** or **6,** respectively. 

#### 3.3.1. 7-(4-Chlorophenyl)-5-methyl-2,3-dihydro-7*H*-thiazolo[3,2-a]pyrimidine-6-carbohydrazide (**5**)

Yield: 66%; 169–170 °C. IR spectrum: 3412, 3325 (NH_2_ and NH), 3065 (CH-aromatic), 2927 (CH-aliphatic), 1655 (C=O), 1605 (C=N). ^1^H-NMR (500 MHz, DMSO-d_6_) δ 2.01 (s, 3H, CH_3_), 3.34 (t, 2H, *J* = 5.8 Hz, CH_2_), 3.41 (t, 2H, *J* = 5.8 Hz, CH_2_), 4.02 (brs, 2H, NH_2_), 4.61 (s, 1H, H-7), 7.56 (d, 2H, *J* = 7.8 Hz, Ar–H), 7.88 (d, 2H, *J* = 7.8 Hz, Ar–H), 10.90 (brs, 1H, NH). ^13^C-NMR (DMSO-d_6_) δ 21.3 (CH_3_), 55.9 (CH_2_), 56.9 (CH_2_), 60.5 (pyrimidine-C), 115.0 (pyrimidine-C), 123.9 (Ar–C), 134.9 (Ar-2C), 140.4 (Ar-2C), 149.9 (Ar-C), 157.4 (pyrimidine-C), 168.4 (pyrimidine-C), 172.2 (C=O). EI-MS (*m/z*, %): 322 (M^+^, 73). Anal. calcd. for C_14_H_15_ClN_4_OS (322.81): C, 52.09; H, 4.68; N; 17.36. Found: C, 52.27; H; 4.59; N; 17.51.

#### 3.3.2. 5-Methyl-7-(thiophen-2-yl)-2,3-dihydro-7*H*-thiazolo[3,2-a]pyrimidine-6-carbohydrazide (**6**)

Yield: 76%; 162–163 °C. IR spectrum: 3425–3390 (NH_2_ and NH), 3060 (CH-aromatic), 2925 (CH-aliphatic), 1660 (C=O), 1610 (C=N). ^1^H-NMR (500 MHz, DMSO-d_6_) δ 1.65 (s, 3H, CH_3_), 3.20 (t, 2H, *J* = 5.8 Hz, CH_2_), 3.50 (t, 2H, *J* = 5.8 Hz, CH_2_), 3.89-4.15 (brs, 3H, NH_2_, H-7), 7.15 (m, 1H, thienyl-H), 7.40 (d, 1H, *J* = 7.2 Hz, thienyl-H), 7.60 (d, 1H, *J* = 6.8 Hz, thienyl-H), 8.49 (brs, 1H, NH). ^13^C-NMR (DMSO-d_6_) δ 22.3 (CH_3_), 55.6 (CH_2_), 57.7 (CH_2_), 79.7 (pyrimidine-C), 120.2 (pyrimidine-C), 121.9 (thienyl-C5), 132.9 (thienyl-C3), 154.4 (thienyl-C4), 154.9 (thienyl-C2), 157.5 (pyrimidine-C), 158.2 (pyrimidine-C), 172.4 (C=O). EI-MS (m/z, %): 294 (M^+^, 70). Anal. calcd. for C_12_H_14_N_4_OS_2_ (294.39): C, 48.96; H; 4.79, N; 19.03. Found: C, 49.07; H, 4.71; N, 19.17.

### 3.4. Sugar-5-(aryl)-7-methyl-2,3-dihydro-5H-thiazolo[3,2-a]pyrimidine-6-carbohydrazone (***7***–***10***)

General procedure: a solution of the acyl hydrazide **5** or **6** (10 mmol) in ethanol (10 mL) was added to a solution of d-galactose or d-xylose (10 mmol) in water (2 mL) followed by addition of glacial acetic acid (0.2 mL). The reaction mixture was heated at reflux temperature for 5 h and then the solvent was removed under reduced pressure. Dry diethyl ether was added to the remaining residue with stirring for 15 min and the formed product was washed with dry ethanol then dried to give the sugar hydrazone derivatives **7**–**10**.

#### 3.4.1. d-Galactose 7-(4-chlorophenyl)-5-methyl-2,3-dihydro-7*H*-thiazolo[3,2-a]pyrimidine-6-carbohydrazone (**7**)

Yield: 71%; brownish foam; IR spectrum: 3425–3421 (OH), 3070 (CH-aromatic), 2930 (CH-aliphatic), 1645 (C=O), 1626 (C=N). ^1^H-NMR (500 MHz, DMSO-*d*_6_) δ 2.40 (s, 3H, CH_3_), 3.36 (t, 2H, *J* = 5.6 Hz, CH_2_), 3.40–3.49 (m, 4H, CH_2_, H-6′,6″), 3.60–3.63 (m, 1H, H-5′), 3.89–4.05 (m, 2H, *H*-4′,3′), 4.65–4.71 (m, 2H, H-2′, OH), 5.15–5.19 (m, 1H, OH), 5.30–5.33 (m, 1H, OH), 5.40–5.44 (m, 1H, OH), 5.60–5.66 (m, 2H, OH, H-7), 7.45 (d, 1H, *J* = 8.5 Hz, H-1′), 7.60 (d, 2H, *J* = 8.2 Hz, Ar–H), 7.88 (d, 2H, *J* = 8.2 Hz, Ar-H), 9.02 (s, 1H, NH). ^13^C-NMR (DMSO-*d*_6_) δ 18.8 (CH_3_), 45.1 (CH_2_), 55.4 (CH_2_), 60.6 (pyrimidine-C), 64.7 (C6′), 70.7 (C5′), 75.1 (C4′), 77.1 (C3′), 82.9 (C2′), 121.4 (pyrimidine-C), 121.9 (Ar-C), 126.9 (Ar-2C), 127.9 (Ar-2C), 139.4 (Ar-C), 140.6 (pyrimidine-C), 141.5 (C1′), 158.0 (pyrimidine-C), 167.9 (C=O). Anal. calcd. for C_20_H_25_ClN_4_O_6_S (484.95): C, 49.53; H, 5.20; N, 11.55. Found: C; 49.37; H; 5.28; N; 11.41.

#### 3.4.2. d-Xylose 7-(4-chlorophenyl)-5-methyl-2,3-dihydro-7*H*-thiazolo[3,2-a]pyrimidine-6-Carbohydrazone (**8**)

Yield: 62%; brownish foam; IR spectrum: 3420–3416 (OH), 3060 (CH-aromatic), 2922 (CH-aliphatic), 1665 (C=O), 1612 (C=N). ^1^H-NMR (500 MHz, DMSO-d_6_) δ 2.38 (s, 3H, CH_3_), 3.37 (t, 2H, *J* = 5.6 Hz, CH_2_), 3.52-3.65 (m, 4H, CH_2_, H-5′,5″), 3.92-4.05 (m, 2H, *H*-4′,3′), 4.67–4.72 (m, 2H, H-2′, OH), 5.15–5.18 (m, 1H, OH), 5.29–5.40 (m, 2H, 2OH), 5.60 (s, 1H, H-7), 7.40 (d, 1H, *J* = 8.5 Hz, H-1′), 7.60 (d, 2H, *J* = 8.2 Hz, Ar–H), 7.88 (d, 2H, *J* = 8.2 Hz, Ar-H), 9.02 (s, 1H, NH). ^13^C-NMR (DMSO-d_6_) δ 18.8 (CH_3_), 45.1 (CH2), 55.4 (CH_2_), 62.6 (pyrimidine-C), 64.5 (C5′), 72.7 (C4′), 77.1 (C3′), 85.9 (C2′), 121.4 (pyrimidine-C), 121.9 (Ar–C), 126.9 (Ar-2C), 127.9 (Ar-2C), 139.4 (Ar-C), 140.6 (pyrimidine-C), 141.5 (C1′), 158.0 (pyrimidine-C), 167.9 (C=O). Anal. calcd. for C_19_H_23_ClN_4_O_5_S (454.93): C, 50.16; H; 5.10, N; 12.32. Found: C, 50.41; H, 5.03; N, 12.15.

#### 3.4.3. d-Galactose 5-methyl-7-(thiophen-2-yl)-2,3-dihydro-7*H*-thiazolo[3,2-a]pyrimidine-6-carbohydrazone (**9**)

Yield: 75%; brownish foam. IR spectrum: 3417–3413 (OH), 3074 (CH-aromatic), 2923 (CH-aliphatic), 1650 (C=O), 1605 (C=N). ^1^H-NMR (500 MHz, DMSO-d_6_) δ 2.30 (s, 3H, CH_3_), 3.40 (t, 2H, *J* = 5.6 Hz, CH_2_), 3.46–3.52 (m, 4H, CH_2_, H-6′,6‴), 3.60–3.63 (m, 1H, H-5′), 3.94-4.10 (m, 2H, *H*-4′,3′), 4.68–4.72 (m, 2H, H-2′, OH), 5.18–5.21 (m, 1H, OH), 5.29–5.33 (m, 1H, OH), 5.39–5.43 (m, 1H, OH), 5.55–5.61 (m, 2H, OH, H-7), 7.35–742 (m, 2H, H-1′, thienyl-H), 7.40 (d, 1H, *J* = 7.2 Hz, thienyl-H), 7.6 (d, 1H, *J* = 6.8 Hz, thienyl-H), 8.92 (brs, 1H, NH). ^13^C NMR (DMSO-d_6_) δ 18.8 (CH_3_), 45.1 (CH_2_), 55.4 (CH_2_), 60.6 (pyrimidine-C), 64.7 (C6′), 70.7 (C5′), 75.1 (C4′), 77.1 (C3′), 82.9 (C2′), 121.4 (pyrimidine-C), 124.9 (thienyl-C5), 126.4 (thienyl-C3), 127.0 (thienyl-C4), 139.4 (thienyl-C2), 140.6 (pyrimidine-C), 141.5 (C1′), 158.0 (pyrimidine-C), 167.9 (C=O). Anal. calcd. for C_18_H_24_N_4_O_6_S_2_ (456.53): C; 47.36; H; 5.30, N, 12.27. Found: C; 47.17; H; 5.42; N; 12.04.

#### 3.4.4. d-Xylose 5-methyl-7-(thiophen-2-yl)-2,3-dihydro-7*H*-thiazolo[3,2-a]pyrimidine-6-Carbohydrazone (**10**)

Yield: 70%; brownish foam. IR spectrum: 3415–3411 (OH), 3055 (CH-aromatic), 2919 (CH-aliphatic), 1655 (C=O), 1523 (C=N). ^1^H-NMR (500 MHz, DMSO-*d*_6_) δ 2.38 (s, 3H, CH_3_), 3.32 (t, 2H, *J* = 5.6 Hz, CH_2_), 3.54–3.66 (m, 4H, CH_2_, H-5′,5‴), 3.95-4.12 (m, 2H, *H*-4′,3′), 4.97–5.11 (m, 3H, H-2′, 2OH), 5.15–5.18 (m, 1H, OH), 5.29–5.40 (m, 2H, OH, H-7), 7.20 (d, 1H, *J* = 8.5 Hz, H-1′), 7.34–7.38 (m, 1H, thienyl-H), 7.41 (d, 1H, *J* = 8.2 Hz, thienyl-H), 7.88 (d, 1H, *J* = 8.2 Hz, thienyl-H), 9.05 (s, 1H, NH). ^13^C-NMR (DMSO-*d*_6_) δ 18.8 (CH_3_), 45.1 (CH_2_), 55.4 (CH_2_), 60.6 (pyrimidine-C), 62.22 (C5′), 70.1 (C4′), 76.1 (C3′), 83.9(C2′), 121.4 (pyrimidine-C), 124.9 (thienyl-C5), 126.4 (thienyl-C3), 127.0 (thienyl-C4), 139.4 (thienyl-C2), 140.6 (pyrimidine-C), 141.5 (C1′), 158.0 (pyrimidine-C), 167.9 (C=O). Anal. calcd. for C_17_H_22_N_4_O_5_S_2_ (426.10): C, 47.87; H; 5.20, N; 13.14. Found: C; 48.02; H; 5.08; N; 13.04.

### 3.5. General Procedure for the Preparation of Compounds (***11***–***14***)

To a solution of the sugar hydrazones **7**–**10** (10 mmol) in pyridine (5 mL), acetic anhydride (3 mL) was added and the mixture was stirred at room temperature for 20 h. The resulting solution was poured onto crushed ice and the product was extracted by ethyl acetate (15 × 3 mL), washed with a saturated solution of sodium hydrogen carbonate (10 mL) followed by water and then the solvent was evaporated to afford the acetylated products **11**–**14**.

#### 3.5.1. Penta-*O*-acetyl-d-galactopentitolyl-7-(4-chlorophenyl)-5-methyl-2,3-dihydro-7*H*-thiazolo[3,2-a]pyrimidine-6-carbohydrazone (**11**)

Yield: 70%; Brownish foam. IR spectrum: 3421 (NH), 3055 (CH-aromatic), 2933 (CH-aliphatic), 1735 (C=O), 1624 (C=N). ^1^H-NMR (500 MHz, DMSO-*d*_6_) δ 2.01 (s, 3H, CH_3_), 2.03 (s, 3H, CH_3_), 2.05 (s, 3H, CH_3_), 2.10 (s, 3H, CH_3_), 2.12 (s, 3H, CH_3_), 2.43 (s, 3H, CH_3_), 3.41–3.45 (m, 4H, 2CH_2_), 3.88–3.95 (m, 2H, H-6′,6″), 4.08–4.16 (m, 1H, H-5′), 4.68–4.80 (m, 2H, H-4′, H-3′), 4.82–4.86 (m, 1H, H-2′), 5.32 (s, 1H, H-7), 7.35 (d, 2H, *J* = 8.4 Hz, Ar–H), 7.41 (d, 2H, *J* = 8.4 Hz, Ar-H), 7.48 (d, 1H, *J* = 7.8 Hz, H-1′), 9.07 (s, 1H, NH). ^13^C-NMR (DMSO-d_6_) δ 18.8, 19.9, 20.2, 20.4, 20.7, 21.1 (6CH_3_), 45.1 (CH_2_), 55.4 (CH_2_), 60.6 (pyrimidine-C), 65.7 (C6′), 72.7 (C5′), 76.14 (C4′), 79.5 (C3′), 83.2(C2′), 121.4 (pyrimidine-C), 121.9 (Ar–C), 126.9 (Ar-2C), 127.9 (Ar-2C), 139.4 (Ar-C), 140.6 (pyrimidine-C), 141.5 (C1′), 158.0 (pyrimidine-C), 167.9, 169.8, 170.1, 170.3, 170.6, 170.8 (6C=O). Anal. calcd. for C_30_H_35_ClN_4_O_11_S (695.14): C; 51.84; H; 5.08, N, 8.06. Found: C; 51.56; H; 5.12; N; 7.92.

#### 3.5.2. Tetra-*O*-acetyl-d-xylotetritolyl-7-(4-chlorophenyl)-5-methyl-2,3-dihydro-7*H*-thiazolo[3,2-a]- pyrimidine-6-carbohydrazone (**12**)

Yield: 61%; Brownish foam. IR spectrum: 3431 (NH), 3048 (CH-aromatic), 2925 (CH-aliphatic), 1749 (C=O), 1630 (C=N). ^1^H-NMR (500 MHz, DMSO-d_6_) δ 2.04 (s, 3H, CH_3_), 2.06 (s, 3H, CH_3_), 2.08 (s, 3H, CH_3_), 2.10 (s, 3H, CH_3_), 2.48 (s, 3H, CH_3_), 2.98 (t, 2H, *J* = 5.8 Hz, CH_2_), 3.25-3.45 (m, 4H, CH_2_, H-5′,5″), 3.90 (m, 1H, H-4′), 4.35–4.58 (m, 2H, H-3′, H-2′), 5.56 (s, 1H, H-7), 7.32 (d, 1H, *J* = 7.6 Hz, H-1′), 7.64 (d, 2H, *J* = 8.4 Hz, Ar-H), 7.78 (d, 2H, *J* = 8.4 Hz, Ar-H), 9.12 (s, 1H, NH). ^13^C-NMR (DMSO-*d*_6_) δ 18.8, 19.8, 20.1, 20.3, 21.2 (5CH_3_), 45.1 (CH_2_), 55.4 (CH_2_), 60.6 (pyrimidine-C), 72.4 (C5′), 75.23 (C4′), 78.5 (C3′), 83.4 (C2′), 121.4 (pyrimidine-C), 121.9 (Ar–C), 126.9 (Ar-2C), 127.9 (Ar-2C), 139.4 (Ar-C), 140.6 (pyrimidine-C), 141.5 (C1′), 158.0 (pyrimidine-C), 168.1, 170.2, 170.4, 170.7, 170.9 (5C=O). Anal. calcd. for C_27_H_31_ClN_4_O_9_S (623.07): C, 52.05; H; 5.02, N; 8.99. Found: C, 51.88; H, 5.09; N, 9.11.

#### 3.5.3. Penta-*O*-acetyl-d-galactopentitolyl-5-methyl-7-(thiophen-2-yl)-2,3-dihydro-7*H*-thiazolo[3,2-a]- pyrimidine-6-carbohydrazone (**13**)

Yield: 74%; Brownish foam. IR spectrum: 3424 (NH), 3066 (CH-aromatic), 2928 (CH-aliphatic), 1740 (C=O), 1615 (C=N). ^1^H-NMR (500 MHz, DMSO-*d*_6_) δ 1.95 (s, 3H, CH_3_), 1.99 (s, 3H, CH_3_), 2.04 (s, 3H, CH_3_), 2.17 (s, 3H, CH_3_), 2.26 (s, 3H, CH_3_), 2.46 (s, 3H, CH_3_), 2.92 (t, 2H, *J* = 5.6 Hz, CH_2_), 3.39–3.60 (m, 4H, CH_2_, H-6′,6″), 4.11–4.15 (m, 1H, H-5′), 4.25-4.34 (m, 2H, H-4′, H-3′), 4.83 (m, 1H, H-2′), 5.34 (s, 1H, H-7), 7.35-7.43 (m, 3H, thienyl-H), 8.05 (d, 1H, *J* = 7.8 Hz, H-1′), 9.12 (brs, 1H, NH). ^13^C-NMR (DMSO-*d*_6_) δ 18.8, 20.1, 20.4, 20.6, 20.9, 21.1 (6CH_3_), 45.1 (CH_2_), 55.4 (CH_2_), 60.6 (pyrimidine-C), 62.7 (C6′), 70.2 (C5′), 74.1 (C4′), 76.1 (C3′), 81.9 (C2′), 121.4 (pyrimidine-C), 124.9 (thienyl-C5), 126.4 (thienyl-C3), 127.0 (thienyl-C4), 139.4 (thienyl-C2), 140.6 (pyrimidine-C), 141.5 (C1′), 158.0 (pyrimidine-C), 167.5, 169.9, 170.2, 170.4, 170.6, 170.9 (6C=O). Anal. calcd. for C_28_H_34_N_4_O_11_S_2_ (666.72): C, 50.44; H, 5.14; N; 8.40. Found: C, 50.21; H; 5.18; N; 8.27.

#### 3.5.4. Tetra-*O*-acetyl-d-xylotetritolyl-5-methyl-7-(thiophen-2-yl)-2,3-dihydro-7*H*-thiazolo[3,2-a]- pyrimidine-6-carbohydrazone (**14**)

Yield: 65%; Brownish foam. IR spectrum: 3407 (NH), 3060 (CH-aromatic), 2928 (CH-aliphatic), 1740 (C=O), 1626 (C=N). ^1^H-NMR (500 MHz, DMSO-*d*_6_) δ 2.01 (s, 3H, CH_3_), 2.03 (s, 3H, CH_3_), 2.05 (s, 3H, CH_3_), 2.11 (s, 3H, CH_3_), 2.44 (s, 3H, CH_3_), 3.39 (t, 2H, *J* = 5.6 Hz, CH_2_), 3.54-3.73 (m, 4H, CH_2_, H-5′,5″), 4.15–4.18 (m, 1H, H-4′), 4.70–4.75 (m, 1H, H-3′), 4.88–4.94 (m, 1H, H-2′), 5.55 (s, 1H, H-7), 7.35–7.38 (m, 1H, thienyl-H), 7.46 (d, 1H, *J* = 7.4 Hz, thienyl-H), 7.55–7.61 (m, 2H, thienyl-H, H-1′), 9.05 (brs, 1H, NH). ^13^C-NMR (DMSO-d_6_) δ 18.8, 19.9, 20.2, 20.4, 20.8 (5CH_3_), 45.1 (CH_2_), 55.4 (CH_2_), 60.6 (pyrimidine-C), 66.2 (C5′), 73.1 (C4′), 76.1 (C3′), 81.9 (C2′), 121.4 (pyrimidine-C), 124.9 (thienyl-C5), 126.4 (thienyl-C3), 127.0 (thienyl-C4), 139.4 (thienyl-C2), 140.6 (pyrimidine-C), 141.5 (C1′), 158.0 (pyrimidine-C), 167.7, 169.9, 170.2, 170.5, 170.9 (5C=O). Anal. calcd. for C_25_H_30_N_4_O_9_S_2_ (594.65): C; 50.50; H; 5.09, N; 9.42. Found: C, 50.35; H; 5.26; N; 9.25.

### 3.6. General Procedure for the Preparation of the Oxadiazoline Substituted Sugar Derivatives (***15***–***18***)

A solution of sugar hydrazones **7**–**10** (10 mmol) in acetic anhydride (15 mL) was heated at 100 °C with stirring for 1.5 h. The resulting solution was poured onto crushed ice, and the product was extracted by ethyl acetate, washed with a solution of sodium hydrogen carbonate followed by water and then dried after evaporation of ethyl acetate. The product was washed two times with diethyl ether–pet. ether mixture (1:1) then dried to give compounds **15**–**18**.

#### 3.6.1. 1-(5-(7-(4-Chlorophenyl)-5-methyl-2,3-dihydro-7*H*-thiazolo[3,2-a]pyrimidin-6-yl)-2-(penta-*O*-acetyl-d-galactopentitolyl)-1,3,4-oxadiazol-3(2*H*)-yl)ethan-1-one (**15**)

Yield: 72%; Brownish foam. IR spectrum: 3048 (CH-aromatic), 2962 (CH-aliphatic), 1735 (C=O), 1675 (C=O), 1618 (C=N). ^1^H-NMR (500 MHz, DMSO-*d*_6_) δ 2.30 (s, 3H, CH_3_), 2.32 (s, 3H, CH_3_), 2.34 (s, 3H, CH_3_), 2.40 (s, 3H, CH_3_), 2.41 (s, 3H, CH_3_), 2,42 (s, 3H, CH_3_), 2.43 (s, 3H, CH_3_), 3.41 (t, 2H, *J* = 6.2 Hz, CH_2_), 3.67–3.88 (m, 4H, CH_2_, H-5′,5″), 4.38–4.42 (m, 1H, H-4′), 4.68–4.72 (m, 1H, H-3′), 4.86-4.92 (m, 1H, H-2′), 4.99-5.04 (m, 1H, H-1′), 5.32 (s, 1H, H-7), 5.70 (d, 1H, *J* = 7.4 Hz, oxadiazoline-H), 7.35 (d, 2H, *J* = 8.4 Hz, Ar-H), 7.41 (d, 2H, *J* = 8.4 Hz, Ar-H). ^13^C-NMR (DMSO-d_6_) 18.6, 19.8, 20.1, 20.7, 21.0, 21.5, 23.4 (7CH_3_), 31.5, 52.8, (2CH_2_), 55.9 (pyrimidine-C), 61.9 (C5′), 62.8 (C4′), 67.7 (C3′), 68.9 (C2′), 75.0 (C1′), 82.5 (oxadiazoline-C), 118.1 (pyrimidine-C), 127.5 (ArC), 128.9 (Ar-2C), 134.5 (Ar-2C), 135.9 (Ar–C), 141.1 (pyrimidine-C), 151.5 (oxadiazoline-C), 158.2 (pyrimidine-C), 169.9, 170.1, 170.4, 170.7, 170.9, 171.2 (6C=O). Anal. calcd. for C_32_H_37_ClN_4_O_12_S (737.17): C, 52.14; H, 5.06; N; 7.60. Found: C, 52.39; H; 5.14; N, 7.79.

#### 3.6.2. 1-(5-(7-(4-Chlorophenyl)-5-methyl-2,3-dihydro-7*H*-thiazolo[3,2-a]pyrimidin-6-yl)-2-(tetra-*O*-acetyl-d-xylotetritolyl)-1,3,4-oxadiazol-3(2*H*)-yl)ethan-1-one (**16**)

Yield: 66%; Brownish foam. IR spectrum: 3053 (CH-aromatic), 2916 (CH), 1735 (C=O), 1680 (C=O) 1616 (C=N). ^1^H-NMR (500 MHz, DMSO-*d*_6_) δ 2.36 (s, 3H, CH_3_), 2.38 (s, 3H, CH_3_), 2.40 (s, 3H, CH_3_), 2.41 (s, 3H, CH_3_), 2.42 (s, 3H, CH_3_), 2.49 ((s, 3H, CH_3_), 3.07 (t, 2H, *J* = 5.8 Hz, CH_2_), 3.43-3.54 (m, 3H, CH_2_, H-4″), 3.92–3.97 (m, 1H, H-4′), 4.35–4.38 (m, 1H, H-3′), 4.77–4.81 (m, 1H, H-2′), 4.90-4.94 (m, 1H, H-1′), 5.35 (s, 1H, H-7), 5.72 (d, 1H, *J* = 7.4 Hz, oxadiazoline-H), 7.29 (d, 2H, *J* = 8.5 Hz, Ar–H), 7.52 (d, 2H, *J* = 8.5 Hz, Ar–H). ^13^C-NMR (DMSO-d_6_) 19.1, 20.5, 20.8, 21.1, 21.5, 23.2 (6CH_3_), 31.4 (CH_2_), 52.7 (CH_2_), 59.30 (pyrimidine-C), 61.8 (C4′), 65.2 (C3′), 68.7 (C2′), 76.4 (C1′), 81.3 (oxadiazoline-C), 118.4 (pyrimidine-C), 128.5 (Ar-C), 130.0 (Ar-2C), 134.4(Ar-2C), 136.0 (Ar-C), 141.0 (pyrimidine-C), 150.7 (oxadiazoline-C), 158.6 (pyrimidine-C), 17.0, 170.3, 170.9, 171.2, 171.5 (5C=O). Anal. calcd. for C_29_H_33_ClN_4_O_10_S (665.11): C, 52.37; H; 5.00, N; 8.42. Found: C, 52.08; H; 5.14; N; 8.31.

#### 3.6.3. 1-(5-(5-Methyl-7-(thiophen-2-yl)-2,3-dihydro-7*H*-thiazolo[3,2-a]pyrimidin-6-yl)-2-(penta-*O*-acetyl-d-galactopentitolyl)-1,3,4-oxadiazol-3(2*H*)-yl)ethan-1-one (**17**)

Yield: 76%; Brownish foam. IR spectrum: 3050 (CH-aromatic), 2929 (CH-aliphatic), 1739 (C=O), 1672 (C=O), 1612 (C=N); ^1^H-NMR (500 MHz, DMSO-*d*_6_) δ 2.30 (s, 3H, CH_3_), 2.32 (s, 3H, CH_3_), 2.36 (s, 3H, CH_3_), 2.39 (s, 3H, CH_3_), 2.43 (s, 3H, CH_3_), 2.46 (s, 3H, CH_3_), 2.49 (s, 3H, CH_3_), 3.23 (t, 2H, *J* = 5.8 Hz, CH_2_), 3.39–3.51 (m, 3H, CH_2_, H-5″), 3.82–4.01 (m, 1H, H-5′), 4.32–4.35 (m, 1H, H-4′), 4.64–4.79 (m, 2H, H-3′, H-2′), 4.98–5.05 (m, 1H, H-1′), 5.21 (s, 1H, H-7), 5.71 (d, 1H, *J* = 7.2 Hz, oxadiazoline-H), 7.81-7.93 (m, 3H, thiophen-H). ^13^C-NMR (DMSO-d_6_) 18.6, 20.1, 20.7, 21.0, 21.4, 21.5, 23.4 (7CH_3_), 31.5 (CH_2_), 52.8 (CH_2_), 40.9 (pyrimidine-C), 61.9 (C5′), 62.8 (C4′), 67.7 (C3′), 68.9 (C2′), 75.0 (C1′), 82.5 (oxadiazoline-C), 118.1 (pyrimidine-C), 127.5 (thienyl-C5),128.9 (thienyl-C3), 136.5 (thienyl-C4), 139.9 (thienyl-C2), 141.1 (pyrimidine-C), 151.5 (oxadiazoline-C), 158.2 (pyrimidine-C), 169.9, 170.1, 170.4, 170.7, 170.9, 171.2 (6C=O). Anal. calcd. for C_30_H_36_N_4_O_12_S_2_ (708.75): C, 50.84; H; 5.12, N; 7.91. Found: C, 50.62; H; 5.04; N; 8.02.

#### 3.6.4. 1-(5-(5-Methyl-7-(thiophen-2-yl)-2,3-dihydro-7*H*-thiazolo[3,2-a]pyrimidin-6-yl)-2-(tetra-*O*-acetyl-d-xylotetritolyl)-1,3,4-oxadiazol-3(2*H*)-yl)ethan-1-one (**18**)

Yield: 68%; Brownish foam. IR spectrum: 3072 (CH-aromatic), 2935 (CH-aliphatic), 1740 (C=O), 1670 (C=O), 1631 (C=N). ^1^H-NMR (500 MHz, DMSO-*d*_6_) δ 2.30 (s, 3H, CH_3_), 2.32 (s, 3H, CH_3_), 2.34 (s, 3H, CH_3_), 2.41 (s, 3H, CH_3_), 2.46 (s, 3H, CH_3_), 2.55 (s, 3H, CH_3_), 3.24–3.27 (m, 4H, 2CH_2_), 3.93–3.97 (m, 1H, H-4″), 4.02–4.08 (m, 1H, H-4′), 4.43–4.47 (m, 1H, H-3′), 4.85–4.88 (m, 1H, H-2′), 5.12–5.17 (m, 2H, H-1′), 5.25 (s, 1H, H-7), 5.70 (d, 1H, *J* = 7.4 Hz, oxadiazoline-H), 7.83–7.92 (m, 3H, thiophen-H). ^13^C-NMR (DMSO-d_6_): 19.1, 20.5, 20.8, 21.1, 21.5, 23.2 (6CH_3_), 31.4 (CH_2_), 52.7 (CH_2_), 47.2 (pyrimidine-C), 61.8 (C4′), 65.2 (C3′), 68.7 (C2′), 76.4 (C1′), 81.3 (oxadiazoline-C), 118.4 (pyrimidine-C), 127.5 (thienyl C-5), 129.5 (thienyl C-3), 134.4 (thienyl C-4), 135.9 (thienyl C-2), 141.0 (pyrimidine-C), 150.7 (oxadiazoline-C), 158.6 (pyrimidine-C), 170.3, 170.5, 170.9, 171.2, 171.5 (5C=O); Anal. calcd. for C_27_H_32_N_4_O_10_S_2_ (636.69): C, 50.93; H; 5.07, N; 8.80. Found: C; 51.05; H; 5.11; N; 8.68.

### 3.7. Materials of the Cell Lines Assay

#### 3.7.1. Cell Culture, Maintenance, and Sub-Culture

Human sensitive and resistant cell lines were purchased from American Type Culture Collection (ATCC, Gaithersburg, MD, USA) as well as human breast cancer MCF7 and MDA-MB-231 cell lines and human colorectal cancer HCT 116 and Caco-2 cell lines. They were cultured using Dulbecco’s modified Eagle’s medium (DMEM) and Roswell Park Memorial Institute (RPMI-1640) medium. All media were supplemented with 4.5g/L glucose w/L-glutamine (Lonza Bioproducts, Belgium) and 10% fetal bovine serum (FBS) (Seralab, UK). The cells were incubated in 5% CO_2_ humidified at 37 °C for growth maintenance.

#### 3.7.2. Cell Proliferation by MTT Assay

The percentages of viable human colorectal cancer HCT 116 and Caco-2 cells as well as human breast cancer MCF7 and MDA-MB-231 cell lines after treatment with different concentrations of the synthesized compounds. These compounds were evaluated by the 3-(4,5-methylthiazol-2-yl)-2,5-diphenyl-tetrazolium bromide (MTT) assay, as reported previously [38], with slight modification. In brief, after evaluation of cell count and viability by trypan blue dye-based method, A549 cells (1 × 10^4^ cells/well) were seeded in a 96 well plate and then kept overnight for attachment. The next day, the complete medium was replaced with fresh one, and then various concentrations of the formulations were investigated on each cell line. After that, cells were allowed to grow for 24 h. Four hours before completion of the incubation period, 10 μL of the MTT (5 mg/mL) was added in each well. After completing the incubation, 100 μL of dimethyl sulfoxide (DMSO) was added to each well and left for 20 min to dissolve the formazan crystals. After the reaction, color development was measured at 450 nm using Bio-Tek microplate reader.

#### 3.7.3. IC_50_ Measurement

The half-maximal inhibitory concentrations (IC_50_) values, which are the concentrations that inhibit 50% of cancer cell viabilities, were obtained by plotting the percentages of cancer cell viabilities versus the concentrations of the sample using polynomial concentration–response curve fitting models (OriginPro 8 software).

## 4. Conclusions

New hybrid compounds of aryl or heteroaryl substituted thiazolopyrimidine system incorporating acyclic sugar moiety derivatives and their derived oxadiazoline compounds were prepared from simple starting compounds. The cytotoxic activities against four cancer cell lines were studied and the prepared compounds that had either an acetylated or deprotected acyclic sugar part were the most active. The results showed the importance of attachment of certain sugar moieties to the thiazolopyrimidine system. The highest activities by the most active candidates were revealed against human colorectal cancer Caco-2 cell lines with the lowest IC_50_ values.

## Figures and Tables

**Table 1:** IC_50_s of the compounds against different colorectal and breast cancerous cell lines.

Compound	HCT116 Cells	Caco-2 Cells	MDA-MB-231 Cells	MCF7 Cells
1	25.28	58.31	40.78	ND
4	63.61	30.84	36.55	ND
8	66.75	9.63	46.99	69.90
9	27.95	ND	ND	ND
10	65.89	4.79	30.58	16.85
11	34.80	83.01	23.35	12.47
13	44.04	16.82	46.30	34.83

IC_50_ values are in µg/mL. ND = IC_50_ undetectable (i.e., IC_50_ more than 100 µg/mL).
